# The Milpa diet: a functional, sustainable pattern for human and planetary health

**DOI:** 10.3389/fnut.2026.1790861

**Published:** 2026-03-13

**Authors:** Rafael Fernández-Demeneghi, Martha Alicia Sánchez-Jiménez, César Huerta-Canseco, Isidro Vargas-Moreno, Gilberto Uriel Rosas-Sánchez, Rodrigo Ramirez-Rodriguez, Gabriela Páez-Huerta, María Magdalena Álvarez-Ramírez

**Affiliations:** 1 Instituto de Investigaciones en Comportamiento Alimentario y Nutrición, Universidad de Guadalajara, Ciudad Guzmán, Mexico; 2 Facultad de Nutrición, Campus Xalapa Universidad Veracruzana, Xalapa, Mexico; 3 Programa de doctorado en Psicología con Orientación en Calidad de Vida y Salud, Universidad de Guadalajara, Ciudad Guzmán, Mexico; 4 Licenciatura en Nutrición, Vicerrectoría de Ciencias de la Salud, Universidad de Monterrey, San Pedro Garza García, Mexico; 5 Departamento de Ciencias de la Tierra y de la Vida, Centro Universitario de Los Lagos, Universidad de Guadalajara, Lagos de Moreno, Mexico; 6 Programa de Estancias Posdoctorales por México, SECIHTI, Centro Universitario de Los Lagos, Universidad de Guadalajara, Lagos de Moreno, Mexico; 7 Instituto Politécnico Nacional, Ciudad de México, Mexico

**Keywords:** agroecology, food sovereignty, indigenous knowledge, Milpa diet, planetary health, precision nutrition

## Introduction: the Milpa diet concept and definition

The synchronization of metabolic dysfunction and planetary degradation constitutes the defining challenge of the Anthropocene. We face a global syndemic—comprising obesity, undernutrition, and climate change—where unhealthy dietary transitions and unsustainable food systems act not merely as consequences, but as a mutually reinforcing feedback loop (1–3). This triple crisis creates a synergistic burden that undermines the viability of sustainable food systems. As environmental and public health degrade, it triggers a regressive cycle where individuals are increasingly subjected to unhealthy food environments, further exacerbating the initial drivers of the syndemic. The magnitude of this crisis is evident: currently, over 2 billion adults present with overweight or obesity (4), while a staggering proportion of mortality attributable to cardiovascular diseases (30%), type 2 diabetes (23%), and chronic kidney disease (20.7%) is directly underpinned by dietary risks (1, 5, 6).

This public health emergency is inextricably linked to an agri-food model dependent on high-yield monocultures. While this industrial paradigm has maximized caloric availability, it has compromised nutritional diversity and ecosystem integrity (7, 8). The expansion of simplified agricultural systems now drives 80% of deforestation and consumes 70% of freshwater (9), generating a “hidden hunger” where caloric abundance coexists with micronutrient deficiencies (10). Consequently, nutrition cannot be approached through a reductionist lens of isolated nutrients; it must be understood as a multidimensional phenomenon shaped by environmental, social, and economic determinants (11, 12).

In this context, the Milpa Diet—rooted in Mesoamerican agricultural traditions—transcends mere cultural grounding to become a robust biocultural system and a complex nutritional ecosystem. Far from being a relic of the past, the Milpa represents a sophisticated, nutrient-dense, and bioactive-rich pattern that aligns with the modern principles of integrative and precision nutrition (13, 14). Historically, dietary interventions have been framed through a reactive, pathogenic lens focused strictly on risk-factor reduction and disease prevention. The Milpa bridges this theoretical gap. Based on the polyculture of maize, beans, squash, and chili, this system certainly supports metabolic health and prevents non-communicable diseases (NCDs) through synergistic biochemical mechanisms rather than isolated compounds (15–17); however, its theoretical grounding goes much further.

**Figure 1:**
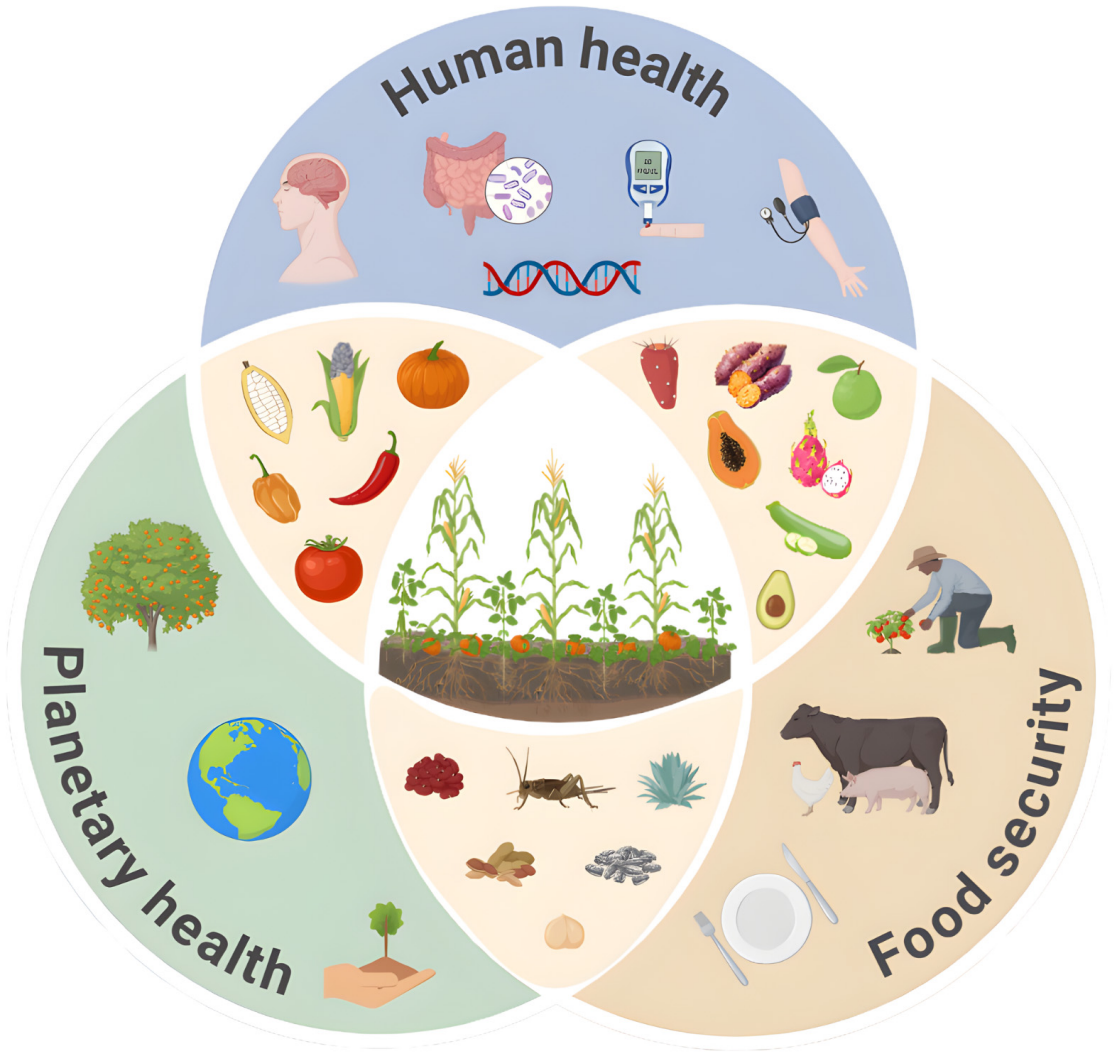
The Milpa paradigm as a synergistic framework for human health, planetary health, and food security. The central intersection represents the core Mesoamerican triad (maize, beans, and squash) cultivated as an agroecological polyculture. The overlapping spheres illustrate the multidimensional impacts of this biocultural system. Human health is supported by diverse, nutrient-dense foods that positively modulate the gut microbiome, metabolic markers, and genomic expression. Planetary health is promoted through biodiversity conservation, sustainable land use, and climate adaptation. Food security is achieved via resilient local agriculture, culturally relevant protein sources (including backyard livestock and edible insects), and food sovereignty. Together, these elements form a holistic, salutogenic nutritional model.

Moving beyond the concept of a mere “meal plan,” the Milpa paradigm embodies a true salutogenic model—focused proactively on the origins of health and the holistic development of wellbeing rather than merely reacting to disease. Its structure fosters food sovereignty, biodiversity preservation, and climate adaptation through efficient resource cycling and agroecological practices (18). This opinion article explores the relevance of the Milpa system not only as a cultural heritage but as a scalable, evidence-based blueprint for sustainable diets (Figure 1). We posit that integrating this indigenous knowledge system offers a strategic, viable opportunity to address the dual burden of chronic disease and environmental degradation, advancing the transition toward resilient, equitable, and sustainable food systems.

## The Milpa system: concept, definition, and nutritional structure

The term Milpa derives from the Nahuatl words *milli* (“sown plot”) and *pan* (“on top of”), designating an ancestral Mesoamerican agricultural system that originated between 4,500- and 3,500-years BP (18, 19). However, functionally, it represents more than a cultivation technique; it is a dynamic biocultural complex adapted to diverse edaphoclimatic conditions. Its nutritional core consists of the “Mesoamerican Triad”: maize (*Zea mays L*.), common bean (*Phaseolus vulgaris L*.), and squash (*Cucurbita pepo L*.), which form a symbiotic structural and nutritional base (20). Depending on regional ecosystems, this polyculture integrates a diverse array of endemic species, functioning as an *in-situ* germplasm bank. These include chili peppers (*Capsicum annuum L*.), tomatoes (*Lycopersicon esculentum*), tomatillo (*Physalis spp*.), and chilacayote (*Cucurbita ficifolia*). The system also incorporates amaranth (*Amaranthus spp*.), nopal cactus (*Opuntia ficus-indica*), and nutrient-dense wild greens known as quelites—such as chipilín (*Crotalaria longirostrata*), *Amaranthus hybridus, Portulaca oleracea*, and *Dysphania ambrosioides*. This biodiversity is complemented by tubers like sweet potato (*Ipomoea batatas*) and cassava (*Manihot esculenta*) (15, 18–21).

### 1 Agroecological synergy and bio-efficiency

From an agroecological perspective, the Milpa thrives as a mixed polyculture within a space of synergistic interactions, optimizing the capture of solar radiation, water, and soil nutrients. The vertical architecture of maize provides support for climbing beans, while the broad leaves of squash cover the soil, retaining moisture and suppressing competitive weeds (15, 19). This structure optimizes the “Land Equivalent Ratio” (LER), resulting in a food system rich in proteins, fiber, vitamins, and bioactive compounds, produced with high resource efficiency (17).

### 2 The technological component: nixtamalization

Crucially, the nutritional architecture of the Milpa diet cannot be understood solely from its raw agricultural products; it relies fundamentally on ancestral food-processing technologies, specifically nixtamalization. This alkaline thermal treatment of maize (cooking with calcium hydroxide) breaks down the hemicellulose of the pericarp, significantly enhancing the bioavailability of niacin (vitamin B3) and calcium, while modifying the protein matrix to improve digestibility (22). Thus, the Milpa system fuses agricultural biodiversity with culinary biotechnology to deliver a complete protein profile and a micronutrient-dense diet.

### 3 The Milpa diet: a functional nutritional matrix

Derived from this agricultural model, the Milpa Diet is proposed not merely as a set of ingredients but as a healthy, sustainable, and culturally relevant dietary pattern. Its nutritional axis relies on the synergistic consumption of maize, beans, squash, and chili (13, 16). These components create a complementary nutritional matrix characterized by a high density of micronutrients, diverse fiber types, and a complex profile of bioactive compounds:

- Maize (*Zea mays*): providing an energy foundation, it provides complex carbohydrates and resistant starch. Enriched by nixtamalization, it becomes a key source of bioavailable calcium. Furthermore, pigmented varieties (blue/red) function as potent reservoirs of anthocyanins—particularly cyanidin-3-glucoside—and phenolic acids (ferulic acid), which are linked to antioxidant and anti-inflammatory pathways (17, 22).
- Common beans (*Phaseolus vulgaris*): these legumes are critical for the protein architecture of the diet. They act as the biochemical complement to maize. In contrast, maize is low in lysine and tryptophan; beans are rich in these essential amino acids, providing protein quality comparable to animal sources. Additionally, they are rich in iron, zinc, folate, and non-digestible fermentable carbohydrates (prebiotics), as well as flavonoids such as quercetin and kaempferol (23, 24).
- Squash (*Cucurbita spp*.): this crop offers a dual nutritional contribution. The seeds (pepitas) provide a dense source of plant-based proteins, zinc, magnesium, and healthy lipids (mono- and polyunsaturated fatty acids), including phytosterols. Conversely, the pulp serves as a primary source of carotenoids (β-carotene, lutein, zeaxanthin), vital for ocular and immune health (25, 26).
- Chili peppers (*Capsicum* spp.): far from being a mere condiment, chili peppers act as metabolic modulators. They are exceptionally rich in Vitamins A, C, and E. Their pungency derives from capsaicinoids, compounds with recognized thermogenic and metabolic-regulating properties, which work in tandem with their high flavonoid content (27).

### 4 Dietary structure and composition

This core triad is dynamically complemented by eight peripheral food groups: vegetables, fruits, whole grains, oilseeds, unsaturated fats, tubers, natural sweeteners, and animal protein in moderation. The regimen prioritizes water consumption while strictly limiting ultra-processed products (16, 17). Collectively, the Milpa Diet constitutes a predominantly plant-based yet fundamentally omnivorous framework. It is defined by a sophisticated macronutrient architecture—rich in plant proteins, complex carbohydrates, and healthy fats—and reinforced by a high fiber content and a diverse phytochemical spectrum.

This combination generates a distinctive nutritional phenotype that modulates metabolic health via multiple bioactive pathways. To illustrate the specific functional contribution of this biodiversity, Table 1 provides a detailed summary of the bioactive compounds and physiological effects associated with the key Mesoamerican species constituting the Milpa Diet.

**Table 1:** Mesoamerican foods included in the Milpa Diet, their bioactive compounds, and health effects.

**Food group/common name (scientific name)**	**Bioactive compounds**	**Health effect**	**Reference**
Maize (*Zea mays* L.)	Polyphenols (ferulic acid), anthocyanins, carotenoids, dietary fiber, phytosterols, linoleic acid (ω-6), oleic acid (ω-9).	Antioxidant, hypocholesterolemic, glycemic control.	(17)
Pumpkin (*Cucurbita pepo* L.)	Phytosterols, cucurbitacins, tocopherols, linoleic acid (ω-6), oleic acid (ω-9), palmitic acid.	Hypocholesterolemic, antioxidant.	(17)
Common bean (*Phaseolus vulgaris* L.)	Polyphenols, flavonoids, saponins, lectins, resistant starch, linoleic acid, α-linolenic acid (ω-3).	Glycemic control, hypocholesterolemic, intestinal health.	(17)
**Vegetables and greens**			
Epazote (*Chenopodium ambrosioides* L.)	Flavonoids, terpenes, polyphenols, fatty acids.	Antiparasitic, antioxidant, anti-inflammatory, immunomodulatory, glycemic control, anticancer	(57)
Habanero pepper (*Capsicum chinense* Jacq.)	Capsaicinoids, carotenoids, polyphenols.	Anti-inflammatory, antioxidant.	(58)
Huanzontle (*Chenopodium nuttalliae*)	Polyphenols, flavonoids, carotenoids.	Anti-inflammatory, antioxidant, hypocholesterolemic.	(59)
Nopal (*Opuntia ficus-indica* L.)	Flavonoids, mucilages, soluble fiber, linoleic acid (ω-6), α-linolenic acid (ω-3).	Anti-inflammatory, antioxidant, glycemic control, antimicrobial, neuroprotective.	(60)
Huitlacoche (*Ustilago maydis*)	β-glucans, polyphenols, linoleic acid (ω-6), oleic acid (ω-9).	Immunomodulatory, antioxidant.	(61)
Tomato (*Solanum lycopersicum* L.)	β-carotene, lycopene, tocopherol, phenolic acids, flavonoids, anthocyanin.	Antioxidant, hypocholesterolemic, antiplatelet aggregation, antithrombotic, antihypertensive, hypoglycemic, anticancer.	(62)
Tomatillo (*Physalis philadelphica* Lam.)	Flavonoids, polyphenols, carotenoids, phytosterols.	Antitumoral, anti-inflammatory.	(63)
Zucchini flower (*Cucurbita pepo* L.)	Flavonoids, carotenoids, polyphenols.	Antioxidant.	(64)
Chayote (*Sechium edule*)	Carotenoids, polyphenols, flavonoids.	Antioxidant, hypocholesterolemic.	(65)
Pepper (*Capsicum annuum* L.)	Capsaicinoids, carotenoids, polyphenols, flavonoids.	Anticancer, anti-inflammatory, antidiabetic, antihypertensive.	(66)
Romeritos (*Suaeda torreyana*)	Polyphenols, carotenoids, fiber, linoleic acid (ω-6).	Antioxidant, hypocholesterolemic.	(67)
Purslane (*Portulaca oleracea*)	Flavonoids, alkaloids, terpenoids, α-linolenic acid (ω-3).	Anti-inflammatory, antioxidant, immunomodulatory.	(68)
**Fruits**			
Black sapote (*Diospyros digyna* Jacq.)	Polyphenols, proanthocyanidins, flavonoids.	Antioxidant.	(69)
Cactus Berry (*Opuntia humifusa*)	Betalains, polyphenols, anthocyanins.	Antioxidant, hypocholesterolemic.	(70)
Canistel (*Pouteria campechiana*)	Carotenoids, polyphenols, flavonoids.	Antioxidant, anti-inflammatory, hypolipidemic, anticancer, glycemic control.	(71)
Cherimoya (*Annona cherimola* Mill.)	Polyphenols, flavanols, procyanidins.	Antioxidant, anticancer, antiallergy.	(72)
Dragon fruit (*Hylocereus undatus*)	Betalains, polyphenols, fiber, anthocyanins, Linoleic acid (ω-6), α-linolenic acid (ω-3).	Antioxidant, hypocholesterolemic, glycemic control, cardioprotective, anti-inflammatory.	(73)
Mamey (*Pouteria sapota*)	Carotenoids, polyphenols.	Antioxidant, cardioprotective, anti-inflammatory, anticancer.	(74)
Mexican hawthorn (*Crataegus mexicana*)	Polyphenols, proanthocyanidins, vitamin C, flavonoids, triterpenic glycosides.	Antioxidant, cardioprotective.	(75)
Papaya (*Carica papaya* L.)	Carotenoids, papain, polyphenols, saponins.	Antioxidant, digestive, anti-inflammatory, antimicrobial, anticancer, glycemic control.	(76)
Pitaya (*Stenocereus stellatus*)	Betalains, polyphenols, flavonoids, fiber.	Antioxidant, hypocholesterolemic, intestinal health, antihypertensive, anti-inflammatory.	(77)
Red mombin (*Spondias purpurea* L.)	Polyphenols, carotenoids, saponins, polysaccharides.	Antioxidant, anti-inflammatory, glycemic control, hypocholesterolemic, ulcer protective, hepatoprotective, antiarthritic, antihypertensive, antiepileptic.	(78)
Sapodilla (*Manilkara zapota*)	Carotenoids, polyphenols.	Antioxidant, anticancer, antimicrobial, anti-inflammatory, antispasmodic, anti-aging, hepatoprotective.	(79)
Xoconostle (*Opuntia joconostle*)	Betalains, flavonoids, polyphenols, fiber, linoleic acid (ω-6), oleic acid (ω-9), tocopherols.	Glycemic control, hypocholesterolemic, antioxidant, anti-inflammatory.	(80)
Soursop (*Annona muricata* L.)	Acetogenins, flavonoids, alkaloids, polyphenols.	Antioxidant, antimicrobial, antinociceptive, antihypertensive, glycemic control, anticancer.	(81)
Yellow mombin (*Spondias mombin* L.)	Polyphenols, flavonoids.	Antioxidant, anti-inflammatory.	(82)
**Whole grains**			
Amaranth (*Amaranthus* spp.)	Squalene, bioactive peptides, polyphenols, oleic acid, linoleic acid, anthocyanins, betalain, carotenoids, flavonoids.	Hypocholesterolemic, gastroprotective, anti-inflammatory, anticancer.	(83)
**Oilseeds and legumes**			
Chia (*Salvia hispanica* L.)	α-Linolenic acid (ω-3), linoleic acid (ω-6), soluble fiber, polyphenols, flavonoids, carotenoids.	Antioxidant, hypotriglyceridemic, hypocholesterolemic, antihypertensive, anticancer, glycemic control.	(84)
Sunflower seed (*Helianthus annuus*)	Polyphenols, phytosterols, flavonoids, carotenoids, linoleic acid (ω-6), oleic acid (ω-9).	Antioxidant, anti-inflammatory, glycemic control, antimicrobial, antihypertensive.	(85)
Pumpkin seed (*Cucurbita pepo*)	Phytosterols, cucurbitacins, tocotrienols, oleic acid (ω-9).	Hypocholesterolemic, antioxidant.	(17)
Cocoa (*Theobroma cacao*)	Flavonoids, epicatechin, proanthocyanidins, saponins, oleic acid (ω-9).	Antioxidant, glycemic control, antihypertensive, anticancer, hypocholesterolemic, neuroprotector.	(86)
Peanut (*Arachis hypogaea*)	Dietary fiber, phytosterols, polyphenols, oleic acid (ω-9), linoleic acid (ω-6), resveratrol, flavonoids.	Hypocholesterolemic, glycemic control, intestinal health, antihypertensive, anticancer, neuroprotector.	(87)
Haba (*Vicia faba* L.)	Polyphenols, flavonoids, fiber, linoleic acid (ω-6), carotenoids.	Glycemic control, antioxidant, intestinal health, antihypertensive, anticancer.	(88)
Lentil (*Lens culinaris* L.)	Polyphenols, flavonoids, tannins, bioactive peptides, linoleic acid (ω-6).	Glycemic control, antioxidant, hypocholesterolemic, anticancer.	(89)
**Unsaturated oils**			
Avocado (*Persea americana* Mill.)	Oleic acid, linoleic acid, polyphenol, phytosterols, carotenoids, tocopherols.	Hypocholesterolemic, antioxidant, cardioprotective.	(90)
**Extra animal protein**			
Grasshopper (*Sphenarium purpurascens*)	Bioactive peptides, linoleic acid (ω-6), α-linolenic acid (ω-3).	Antioxidant, hypocholesterolemic, intestinal health.	(91)
Egg	Lutein, zeaxanthin, monounsaturated, polyunsaturated fatty acids, carotenoids.	Hypocholesterolemic, antioxidant, cardioprotective.	(92)
Chicken	Bioactive peptides, oleic acid (ω-9).	Cardioprotective, glycemic control.	(93)
Fish	α-Linolenic acid (EPA, DHA).	Antioxidant, anti-inflammatory, neuroprotective, cardioprotective, antimicrobial, hepatoprotective.	(94)
**Tubers**			
Yucca (*Manihot esculenta*)	Resistant starch, polyphenols, saponins, flavonoids, tanins, terpenoids.	Antibacterial, antioxidant, glycemic control, anti-inflammatory. anticancer, anti-diarrheal, hypocholesterolemic.	(95)
Sweet potato (*Ipomoea batatas*)	Carotenoids, fiber, polyphenols, anthocyanins, linoleic acid (ω-6).	Antioxidant, glycemic control, cardioprotective, anti-inflammatory, anticancer, antimicrobial.	(96)
**Sweeteners**			
Honey (*Apis mellifera* L.)	Flavonoids, polyphenols, carotenoids, enzymes.	Antioxidant, anti-inflammatory, glycemic control, antimicrobial, antiparasitic, antiviral, anticancer.	(97)

Bioactive components are listed in descending order of content.

## The Milpa diet and health: mechanisms and bioactive architecture

Contemporary nutritional science effectively validates the therapeutic potential of traditional dietary patterns rooted in whole, minimally processed foods. The Milpa diet aligns rigorously with this paradigm, offering a functional food matrix characterized by a low glycemic load, high fiber density, and a diverse spectrum of bioactive compounds. Unlike reductionist approaches, the therapeutic potency of this diet relies on the biochemical synergy of its components—a “natural polypharmacy” effect that targets metabolic dysregulation through multiple signaling pathways (17, 23).

### 1 Mechanisms of action: the matrix effect

Specific food-processing interactions and enzymatic inhibitions drive the physiological impact of the Milpa. For instance, the nixtamalization of maize not only enhances calcium bioavailability but modifies the anthocyanin profile, increasing the concentration of cyanidin-3-glucoside (C3G), a flavonoid with proven insulin-sensitizing properties (28, 29). Concurrently, the consumption of common beans provides specific inhibitors of α-amylase and α-glucosidase. These bioactive peptides blunt postprandial glycemic excursions by delaying carbohydrate hydrolysis (30, 31). When combined with the lipid profile of pumpkin seeds (rich in PUFAs and phytosterols), this triad creates a metabolic buffer that optimizes glucose homeostasis and lipid metabolism.

### 2 Bioactive architecture

The health benefits are mechanistically attributable to a distinct phytochemical profile that exerts pleiotropic effects (23, 32). Key bioactive groups include:

- Phenolic compounds: specifically, flavonoids, tannins, and phenolic acids. These act as potent antioxidants and modulators of inflammatory pathways (e.g., NF-κB inhibition) (23).
- Phytosterols: including β-sitosterol, campesterol, and stigmasterol. These compounds compete with cholesterol for micellar absorption in the gut, effectively improving lipid profiles (23, 33).
- Carotenoids: such as zeaxanthin, lutein, and β-carotene. Beyond their role in ocular health, they function as signaling molecules in adipose tissue regulation (17).
- Bioactive peptides: derived from plant proteins (beans and amaranth), exhibiting antihypertensive (ACE-inhibitory) and antithrombotic activities (34).
- Dietary fiber and fatty acids: the synergy between soluble fiber and oleic/linoleic acids supports satiety signaling and improved insulin receptor sensitivity (35, 36).

### 3 Clinical evidence: from prevention to management

Accumulating evidence demonstrates that adherence to the Milpa diet—and the broader Traditional Mexican Diet (TMD)—confers protection against the spectrum of chronic non-communicable diseases (NCDs). Clinical and epidemiological studies have documented significant risk reductions in:

- Cardiometabolic conditions: including Type 2 Diabetes (T2D), dyslipidemias, Metabolic dysfunction-associated steatotic liver disease (MASLD), and obesity (13, 16, 37–39).
- Systemic pathologies: such as cardiovascular disease (CVD), chronic kidney disease (CKD), and specific cancers, as well as autoimmune conditions like celiac disease (5, 17, 40).

### 4 Precision nutrition and the gut-brain axis

Finally, the Milpa diet represents a frontier for Precision Nutrition. Its plant-forward composition serves as a high-quality substrate for the gut microbiota, promoting the production of Short-Chain Fatty Acids (SCFAs), such as butyrate. This mechanism not only reinforces gut barrier integrity but also modulates the gut-brain axis (41, 42). Crucially, these benefits are amplified by the synergy of functional foods inherent to the system. Beyond providing essential nutrients, the diversity of Milpa crops delivers a complex matrix of bioactive compounds with antioxidant and anti-inflammatory properties that play a key role in mental health (23, 43). As research advances in nutrigenomics, the Milpa stands as a scalable model to address genetic susceptibilities (e.g., MTHFR or ABCA1 variants) through these culturally relevant dietary interventions (13, 44).

## Clinical and epidemiological evidence: moving beyond the western diet

The Western Diet (WD)—characterized by excessive caloric density, nutrient deficiency, and a reliance on ultra-processed foods (UPFs)—acts as a primary driver of metabolic inflexibility and systemic chronic inflammation (2). The public health ramifications of this dietary transition are catastrophic, reflected in the escalating global mortality burden linked to poor nutritional quality (1, 5).

Current epidemiological estimates paint a stark picture of this “metabolic collision.” Approximately 6.58 million deaths from cardiovascular disease (CVD) are attributable to dietary risks, specifically the excessive intake of sodium, trans fats, and processed meats (6). Furthermore, nutritional imbalances—such as low whole-grain and fruit consumption—are directly linked to 381,000 deaths from Type 2 Diabetes (T2D) (1) and 317,000 deaths among patients with chronic kidney disease (CKD) (5). Moreover, the obesity epidemic, fueled by the WD, now accounts for nearly 357,000 cancer deaths annually (45, 46). Extending beyond somatic pathology, this dietary pattern drives neuronal alterations that have opened a critical debate regarding the addictive potential of ultra-processed foods, further underscoring the emerging intersection between nutrition and mental health (47).

Against this backdrop, the Milpa diet emerges not merely as a cultural alternative but as a potent physiological corrective. While clinical literature often categorizes these interventions as “Traditional Mexican Diet” (TMD) or “Genome-Based Diets,” they are fundamentally rooted in the Milpa triad and its associated agrobiodiversity. To systematically validate this, existing studies provide robust evidence across various methodological designs and sample populations:

Cross-sectional epidemiological evidence demonstrates that reintroducing these pre-Hispanic staples generates a favorable lipidomic profile among adult populations, significantly reducing total cholesterol, LDL-c, VLDL-c, and triglycerides—key drivers of atherogenic risk (37, 44). Furthermore, randomized crossover feeding trials in women of Mexican descent reveal that the diet exerts a regulatory effect on glucose homeostasis, reducing insulin levels and HOMA-IR (44). Significantly, these controlled trials show it lowers serum concentrations of high-sensitivity C-reactive protein (hsCRP), thereby dampening the systemic low-grade inflammation characteristic of the WD (48).

Crucially, the Milpa diet exhibits potential as a tool for population-specific precision nutrition. Clinical intervention studies indicate that its metabolic benefits are particularly pronounced in individuals carrying risk polymorphisms common in Amerindian and Mestizo populations (e.g., *MTHFR* 677 CT, *ABCA1* R230C, and *APOE* ε*4*). This suggests that the Milpa pattern may mitigate the deleterious phenotypic expression of these high-risk genotypes (44, 48).

Mechanistically, *in vivo* animal models utilizing rats fed a “Pre-Hispanic Diet” formulation confirm these observations at the molecular level. Consumption of this pattern is linked to the upregulation of key metabolic regulators, including PPAR-α (fatty acid oxidation), UCP-1 (thermogenesis), and the mitochondrial biogenesis coactivator PGC-1α.

This molecular cascade results in reduced adipocyte hypertrophy and lower hepatic triglyceride accumulation (49). Concomitantly, the diet improves the cellular redox state by decreasing Reactive Oxygen Species (ROS) and oxidized proteins while optimizing the GSSG/GSH ratio. Notably, these neuroprotective and antioxidant mechanisms promote systemic conditions associated with cognitive health (43, 49, 50).

## The Milpa diet for planetary health

The current anthropogenic crisis reveals a dangerous paradox: while approximately 82% of the human caloric intake comes from plants (51), global dietary diversity has collapsed. Despite the existence of over 6,000 edible plant species, 60% of agricultural production is effectively monopolized by just nine crops (e.g., maize, wheat, rice, soy). These are produced almost exclusively via high-input monocultures intended not for direct human nutrition but for the manufacture of ultra-processed food ingredients (3, 7). This industrial model drives soil depletion, biodiversity loss, and creates a critical rift between human consumption and environmental sustainability.

In stark contrast, the Milpa system functions as a model of ecological intensification, capable of sustaining productivity even under adverse climatic conditions (20). Unlike monocultures that act as biological vacuums, the Milpa thrives on the specific niche complementarity of the Mesoamerican triad:

- Maize (C4 architecture): acts as the vertical axis, optimizing solar radiation capture via C4 photosynthesis while providing physical support for climbing legumes.
- Beans (nitrogen fixation): establishes a symbiotic relationship with *Rhizobium* bacteria to fix atmospheric nitrogen, naturally refertilizing the soil and enriching the system for associated crops.
- Squash (microclimate regulation): its broad leaves function as a living mulch, intercepting sunlight to suppress competitive weeds, maintaining soil moisture, and secreting cucurbitacins—biochemical compounds that provide allelopathic protection against pests (19, 20, 52).

This biological synergy generates a robust microclimate that optimizes the “Land Equivalent Ratio” (LER). The diverse aerial architecture and complex root stratification enable efficient resource partitioning, significantly enhancing resilience against droughts and pest outbreaks compared with simplified systems (18, 19).

Consequently, the Milpa stands as an agroecological system that embodies the principles of Planetary Health, effectively linking sustainable food production with ecosystem conservation and human wellbeing (15, 53). Its historical persistence serves as a rigorous proof of concept: it demonstrates that it is possible to harmonize nutritional needs with planetary boundaries, offering a sustainability model rooted in traditional knowledge and respectful coexistence with nature.

Beyond its response to routine anthropogenic impacts, the systemic stability of the Milpa positions it as a critical nutritional solution during climate-induced emergencies and natural disasters. As global, highly centralized supply chains become increasingly vulnerable to severe climate shocks, the reliance on imported ultra-processed foods poses a severe threat to regional food security. Developing and safeguarding culture-specific dietary profiles like the Milpa serves as a vital contingency strategy. By maintaining local agricultural knowledge and utilizing indigenous, drought-resistant crops, communities establish a reliable, nutrient-dense safety net when industrial food systems fail. In this light, the preservation of local culinary heritage transcends cultural identity; it becomes a tangible, life-saving strategy for food security and disaster resilience in an era of climate unpredictability.

## Discussion: challenges, opportunities, and the strategic revaluation of the Milpa

While the physiological and ecological evidence supporting the Milpa diet is compelling, its effective reimplementation faces systemic barriers rooted in the “industrial lock-in” of current food systems. The globalization of agriculture has homogenized dietary landscapes, creating environments where ultra-processed foods are ubiquitous and often cheaper than fresh staples (7, 8). However, this transition has created a critical dissonance: while globalization offers dietary diversity, nutrition must not be governed by transient trends. The definition of a “correct diet” remains immutable in its biological principles—complete, varied, sufficient, safe, and adequate—but in the Anthropocene, it must imperatively include two new dimensions: sustainability and cultural pertinence.

In this context, the Milpa diet represents the closest approximation to population-based precision nutrition. While global frameworks like the EAT-Lancet planetary health diet provide vital universal targets for sustainable eating, and the Mediterranean diet serves as a recognized gold standard for cardiometabolic health, the Milpa diet offers a critical localized counterpart. Universal models, while highly valuable, often lack the cultural resonance and specific genomic synchronization required for diverse populations. For instance, while the Mediterranean diet achieves cardiovascular protection largely through olive oil and wine, the Milpa achieves comparable functional synergy through the lipid profiles of pumpkin seeds, avocados, and the bioactive peptides of diverse legumes.

For Mesoamerican and Mestizo populations, adopting the Milpa pattern is not merely a cultural choice but a biological imperative; it respects the genetic architecture of native peoples, offering a metabolic substrate to which they are historically and evolutionarily adapted (44, 54). Unlike imported dietary models that may exacerbate metabolic discordance, the Milpa offers a “genomic synchronization” that valorizes ancestral wisdom not just as folklore, but as empirically valid preventive medicine.

It is important to note the contextual boundaries of this system. The exact crop matrix of the Milpa is highly scalable and therapeutically optimal within Mesoamerica and for Hispanic/Latine populations globally. However, its direct agricultural implementation may not be ecologically applicable or culturally resonant in fundamentally different edaphoclimatic zones (e.g., Northern Europe or Arid Asia). In such contexts, the Milpa serves not as a literal dietary export, but as a conceptual paradigm. It demonstrates how reclaiming any region's native, agroecological polycultures (e.g., the Nordic diet or traditional Asian intercropping systems) can simultaneously resolve local health and environmental crises.

Adopting this pattern generates a virtuous cycle of nutritional and agroecological synergy. By consuming local polycultures, we amplify functionality: the diet becomes more bioactive, while the land becomes more resilient. This approach transcends the reductionist view of nutrition as mere “food intake,” repositioning it as a multidimensional phenomenon encompassing culture, society, the economy, health, and prevention. It fosters local economies, strengthens food sovereignty, and dignifies the rural sector, countering the stigmatization that has marginalized indigenous foodways (14, 18, 55).

To operationalize this salutogenic vision—focused on generating health rather than managing disease—scientific knowledge must transcend the laboratory. Effective science communication serves as the critical bridge to translate these findings into robust public policies. We propose a systemic intervention framework:

1. Agroecological policy reorientation: shift subsidies toward polycultural, small-scale farming to conserve native germplasm *in-situ* and ensure the resilience of the diet's genetic base (51).
2. From superfoods to biocultural heritage: educational strategies must dismantle the “superfood” narrative (which leads to commodification and elitism) and replace it with a “Biocultural Heritage” model. This validates indigenous food systems as scientifically superior strategies for community health (15).
3. Sustainable public procurement: incorporating Milpa foods into school feeding programs and public institutions is a potent tool for normalizing these nutrient-dense foods, ensuring that the “correct diet” is accessible to the most vulnerable.
4. Mindful eating as a promotion strategy: since “real food” lacks the massive advertising machinery of ultra-processed products, we must actively cultivate Mindful Eating not merely as a clinical tool, but as a holistic public health necessity. This approach reawakens the conscious connection between the eater, the origin of the food, and the act of nourishment (56). By emphasizing the sensory and cultural richness of the Milpa, we can counter the industrial marketing narrative and empower individuals to intentionally choose health and sustainability over convenience.

Ultimately, the revitalization of the Milpa diet demands treating individual health, community well-being, and planetary sustainability as indivisible outcomes.

## Conclusion: a path toward sustainable health and resilience

The Milpa diet transcends the traditional meal concept to become a sophisticated, scalable strategy for planetary health. By synchronizing human metabolic needs—specifically adhering to the genetic adaptations of Mesoamerican populations—with agroecological resilience, it effectively resolves the dichotomy between nutrition and sustainability. Revitalizing this system is not an exercise in nostalgia, but a critical innovation for the Anthropocene. Ultimately, the Milpa offers a proven, salutogenic blueprint to navigate the global syndemic, demonstrating that the most resilient future for nutrition lies in the scientific revalorization of our ancestral roots.

## References

[1] Ma H Wang M Qin C Shi Y Mandizadza OO Ni H . Trends in the burden of chronic diseases attributable to diet-related risk factors from 1990 to 2021 and the global projections through 2030: a population-based study. Front Nutr. (2025) 12:1570321. doi: 10.3389/fnut.2025.1570321 40416367 PMC12098078

[2] Clemente-Suárez VJ Beltrán-Velasco AI Redondo-Flórez L Martín-Rodríguez A Tornero-Aguilera JF. Global impacts of western diet and its effects on metabolism and health: a narrative review. Nutrients. (2023) 15:2749. doi: 10.3390/nu15122749 37375654 PMC10302286

[3] Vega-Mejía N Ponce-Reyes R Martinez Y Carrasco O Cerritos R. Implications of the western diet for agricultural production, health and climate Change. Front Sustain Food Syst. (2018) 2:88. doi: 10.3389/fsufs.2018.00088

[4] GBD 2021 Adolescent BMI Collaborators. Global, regional, and national prevalence of child and adolescent overweight and obesity, 1990–2021, with forecasts to 2050: a forecasting study for the Global Burden of Disease Study 2021. Lancet. (2025) 405:785–812. doi: 10.1016/S0140-6736(25)00397-6 40049185 PMC11920006

[5] Wei N Yang M Zheng P Xu J. Burden and inequalities of chronic kidney disease attributable to diet globally, regionally and temporally, 1990-2021. Front Nutr. (2025) 12:1592389. doi: 10.3389/fnut.2025.1592389 40607040 PMC12213357

[6] Vaduganathan M Mensah GA Turco JV Fuster V Roth GA. The global burden of cardiovascular diseases and risk: a compass for future health. J Am Coll Cardiol. (2022) 80:2361–71. doi: 10.1016/j.jacc.2022.11.005 36368511

[7] Leite FHM Khandpur N Andrade GC Anastasiou K Baker P Lawrence M . Ultra-processed foods should be central to global food systems dialogue and action on biodiversity. BMJ Glob Health. (2022) 7:e008269. doi: 10.1136/bmjgh-2021-00826912 35346976 PMC8895941

[8] Fardet A Rock E. Ultra-processed foods and food system sustainability: what are the links? Sustainability. (2020) 12:6280. doi: 10.3390/su12156280

[9] United Nations Convention to Combat Desertification (UNCCD). Global Land Outlook 2: Land Restoration for Recovery and Resilience. Bonn: UNCCD. (2022). Available online at: https://www.unccd.int/sites/default/files/2022-04/UNCCD_GLO2_low-res_2.pdf (Accessed January 2, 2026).

[10] Fanzo J Rudie C Sigman I Grinspoon S Benton TG Brown ME . Sustainable food systems and nutrition in the 21st century: a report from the 22nd annual harvard nutrition obesity symposium. Am J Clin Nutr. (2022) 115:18–33. doi: 10.1093/ajcn/nqab315 34523669 PMC8755053

[11] Aydogdu GS Karadag MG. The two dimensions of nutrition for the planet: environment and health. Curr Nutr Rep. (2025) 14:49. doi: 10.1007/s13668-025-00642-3 40111708 PMC11926033

[12] Sparling TM Offner C Deeney M Denton P Bash K Juel R . Intersections of climate change with food systems, nutrition, and health: an overview and evidence map. Adv Nutr. (2024) 15:100274. doi: 10.1016/j.advnut.2024.10027448 39019218 PMC11382032

[13] Huerta-Álvarez A Arellano M Chávez-Méndez CA Carpinteyro-Espin P Palacios-Reyes C Pérez-Escobar J. Milpa diet for MASLD in mesoamerican populations: feasibility, advantages, and future perspectives. Life. (2025) 15:812. doi: 10.3390/life15050812 40430238 PMC12113525

[14] Mota-Cruz C Casas A Ortega-Paczka R Perales H Vega-Peña E Bye R. Milpa, a long-standing polyculture for sustainable agriculture. Agriculture. (2025) 15:1737. doi: 10.3390/agriculture15161737

[15] Benrey B Bustos-Segura C Grof-Tisza P. The mesoamerican milpa system: Traditional practices, sustainability, biodiversity, and pest control. Biol Control. (2024) 198:105637. doi: 10.1016/j.biocontrol.2024.105637

[16] Biruete A Leal-Escobar G Espinosa-Cuevas Á Mojica L Kistler BM. Dieta de la Milpa: a culturally-concordant plant-based dietary pattern for hispanic/latine people with chronic kidney disease. Nutrients. (2024) 16:574. doi: 10.3390/nu16050574 38474703 PMC10934134

[17] Sánchez-Velázquez OA Luna-Vital DA Morales-Hernández N Contreras J Villaseñor-Tapia EC Fragoso-Medina JA . Nutritional, bioactive components and health properties of the Milpa triad system seeds (corn, common bean and pumpkin). Front Nutr. (2023) 10:1169675. doi: 10.3389/fnut.2023.1169675 37538927 PMC10395131

[18] Vazeux-Blumental N Manicacci D Tenaillon M. The milpa, from Mesoamerica to present days, a multicropping traditional agricultural system serving agroecology. C R Biol. (2024) 347:159–73. doi: 10.5802/crbiol.164 39503997

[19] Zizumbo-Villarreal D Flores-Silva A Colunga-García Marín P. The Archaic diet in Mesoamerica: incentive for Milpa development and species domestication. Econ Bot. (2012) 66:328–43. doi: 10.1007/s12231-012-9212-5

[20] Fonteyne S Castillo Caamal JB Lopez-Ridaura S Van Loon J Balbuena JE Osorio Alcalá L . Review of agronomic research on the Milpa, the traditional polyculture system of Mesoamerica. Front Agron. (2023) 5:1115490. doi: 10.3389/fagro.2023.1115490

[21] Rojas-Sánchez B. de los Santos-Villalobos S, Valdez Alarcón JJ, Chávez-Bárcenas AT, Orozco-Mosqueda MC, Santoyo G. Optimizing milpa agrosystems with beneficial microbes and their ecological interactions: a review. Discover Appl Sci. (2025) 7:104. doi: 10.1007/s42452-025-06503-6

[22] Odukoya JO De Saeger S De Boevre M Adegoke GO Audenaert K Croubels S . Influence of nixtamalization cooking ingredients on the minerals composition of nixtamalized maize and sorghum. J Cereal Sci. (2022) 103:103373. doi: 10.1016/j.jcs.2021.103373

[23] Méndez-Flores OG Ochoa-Díaz López H Castro-Quezada I Olivo-Vidal ZE García-Miranda R Rodríguez-Robles U . The Milpa as a supplier of bioactive compounds: a review. Food Rev Int. (2023) 39:1359–76. doi: 10.1080/87559129.2021.1934001

[24] Celmeli T Sari H Canci H Sari D Adak A Eker T . The nutritional content of common bean (*Phaseolus vulgaris* L.) landraces in comparison to modern varieties. Agronomy. (2018) 8:166. doi: 10.3390/agronomy8090166

[25] Huerta-Reyes M Tavera-Hernández R Alvarado-Sansininea JJ Jiménez-Estrada M. Selected species of the Cucurbitaceae family used in Mexico for the treatment of diabetes mellitus. Molecules. (2022) 27:3440. doi: 10.3390/molecules27113440 35684376 PMC9182361

[26] Adnan M Gul S Batool S Fatima B Rehman A Yaqoob S . A review on the ethnobotany, phytochemistry, pharmacology and nutritional composition of *Cucurbita pepo* L. J Phytopharmacol. (2017) 6:133–9. doi: 10.31254/phyto.2017.6211

[27] Hernandez-Perez T Gómez-García MR Valverde ME Paredes-López O. Capsicum annuum (hot pepper): an ancient Latin-American crop with outstanding bioactive compounds and nutraceutical potential. Compr Rev Food Sci Food Saf. (2020) 19:2972–93. doi: 10.1111/1541-4337.12634 33337034

[28] Mora-Rochín S Gaxiola-Cuevas N Gutiérrez-Uribe JA Milán-Carrillo J Milán-Noris EM Reyes-Moreno C . Effect of traditional nixtamalization on anthocyanin content and profile in Mexican blue maize (*Zea mays* L.) landraces. LWT-Food Sci Technol. (2016) 68:563–9. doi: 10.1016/j.lwt.2016.01.009

[29] Martín MÁ Ramos S. Dietary flavonoids and insulin signaling in diabetes and obesity. Cells. (2021) 10:1474. doi: 10.3390/cells10061474 34208379 PMC8231211

[30] Oseguera-Toledo ME de Mejia EG Amaya-Llano SL. Hard-to-cook bean (*Phaseolus vulgaris* L.) proteins hydrolyzed by alcalase and bromelain produced bioactive peptide fractions that inhibit targets of type-2 diabetes and oxidative stress. Food Res Int. (2015) 76:839–51. doi: 10.1016/j.foodres.2015.07.046 28455070

[31] Mojica L Chen K de Mejía EG. Impact of commercial precooking of common bean (*Phaseolus vulgaris*) on the generation of peptides, after pepsin–pancreatin hydrolysis, capable to inhibit dipeptidyl peptidase-IV. J Food Sci. (2015) 80:H188–98. doi: 10.1111/1750-3841.12726 25495131

[32] Almaguer González JA García Ramírez HJ Vargas Vite V Padilla Mirazo M. Fortalecimiento de la Salud Con Comida, Ejercicios y Buen Humor: La Dieta de la Milpa Modelo de Alimentación Mesoamericana Saludable y Culturalmente Pertinente. Ciudad de México: Secretaría de Salud (2018). Available at: https://pesquisa.bvsalud.org/portal/resource/pt/biblio-880586

[33] Ríos-Hoyo A Romo-Araiza A Meneses-Mayo M Gutiérrez-Salmeán G. Prehispanic functional foods and nutraceuticals in the treatment of dyslipidemia associated to cardiovascular disease: a mini-review. Int J Vitam Nutr Res. (2017) 87:85–98. doi: 10.1024/0300-9831/a000290 28128718

[34] Hernández-Pérez T Paredes-López O. Ancient Latin-American food crops: an overview of their nutraceutical and antiobesity peptides. Food Sci Tech Int. 2025;0(0). doi: 10.1177/10820132251319934 39981629 PMC13144648

[35] López-Romero P Pichardo-Ontiveros E Avila-Nava A Vázquez-Manjarrez N Tovar AR Pedraza-Chaverri J . The effect of nopal (*Opuntia ficus indica*) on postprandial blood glucose, incretins, and antioxidant activity in Mexican patients with type 2 diabetes after consumption of two different composition breakfasts. J Acad Nutr Diet. (2014) 114:1811–8. doi: 10.1016/j.jand.2014.06.352 25132122

[36] Alatorre-Cruz JM Carreño-López R Alatorre-Cruz GC Paredes-Esquivel LJ Santiago-Saenz YO Nieva-Vázquez A. Traditional Mexican food: phenolic content and public health relationship. Foods. (2023) 12:1233. doi: 10.3390/foods12061233 36981159 PMC10048498

[37] Valerino-Perea S Armstrong MEG Papadaki A. Adherence to a traditional Mexican diet and non-communicable disease-related outcomes: secondary data analysis of the cross-sectional Mexican national health and nutrition survey. Br J Nutr. (2023) 129:1266–79. doi: 10.1017/S0007114522002331 35876036 PMC10011591

[38] Santiago-Torres M Kratz M Lampe JW Tapsoba JD Breymeyer KL Levy L . Metabolic responses to a traditional Mexican diet compared with a commonly consumed US diet in women of Mexican descent: a randomized crossover feeding trial. Am J Clin Nutr. (2016) 103:366–74. doi: 10.3945/ajcn.115.119016 26718418 PMC4733259

[39] Loroña NC Santiago-Torres M Lopez-Pentecost M . Traditional Mexican dietary pattern and cancer risk among women of Mexican descent. Cancer Causes Control. (2024) 35:887–96. doi: 10.1007/s10552-024-01849-5 38305935 PMC11129927

[40] Lopez-Pentecost M Tamez M Mattei J Jacobs ET Thomson CA Garcia DO. Adherence to a traditional mexican diet is associated with lower hepatic steatosis in US-born hispanics of Mexican descent with overweight or obesity. Nutrients. (2023) 15:4997. doi: 10.3390/nu15234997 38068856 PMC10708445

[41] Guevara-Cruz M Tovar AR Aguilar-Salinas CA Medina-Vera I Gil-Zenteno L Hernández-Viveros I . A dietary pattern including nopal, chia seed, soy protein, and oat reduces serum triglycerides and glucose intolerance in patients with metabolic syndrome. J Nutr. (2012) 142:64–9. doi: 10.3945/jn.111.147447 22090467

[42] Guizar-Heredia R Noriega LG Rivera AL Resendis-Antonio O Guevara-Cruz M Torres N . A new approach to personalized nutrition: postprandial glycemic response and its relationship to gut microbiota. Arch Med Res. (2023) 54:176–88. doi: 10.1016/j.arcmed.2023.02.007 36990891

[43] Fernández-Demeneghi R Domínguez-Pantoja M Martínez-Moreno AG Vargas-Moreno I Ramirez-Rodriguez R. Positioning berries in nutritional psychiatry: potential for prevention and co-therapy in mental health. Front Behav Neurosci. (2025) 19:1622242. doi: 10.3389/fnbeh.2025.1622242 41064748 PMC12500709

[44] Ojeda-Granados C Panduro A Rivera-Iñiguez I Sepúlveda-Villegas M Roman S. A regionalized genome-based Mexican diet improves anthropometric and metabolic parameters in subjects at risk for obesity-related chronic diseases. Nutrients. (2020) 12:645. doi: 10.3390/nu12030645 32121184 PMC7146143

[45] Figlioli G Piovani D Tsantes AG Pugliese N Nikolopoulos GK Hassan C . Burden of cancer attributable to high body mass index: a systematic analysis of the global burden of disease study 2021. Clin Nutr. (2025) 48:144–52. doi: 10.1016/j.clnu.2025.04.002 40215883

[46] Li X Li H. Global, national, and regional burden of cancer attributable to dietary risk: results from the global burden of disease study 2021. BMC Public Health. (2025) 25:3244. doi: 10.1186/s12889-025-24570-7 41034906 PMC12487502

[47] Gearhardt AN DiFeliceantonio AG. Highly processed foods can be considered addictive substances based on established scientific criteria. Addiction. (2023) 118:589–98. doi: 10.1111/add.16065 36349900

[48] Santiago-Torres M Tinker LF Allison MA Breymeyer KL Garcia L Kroenke CH . Development and use of a Traditional Mexican Diet Score in relation to systemic inflammation and insulin resistance among women of Mexican descent. J Nutr. (2015) 145:2732–40. doi: 10.3945/jn.115.21353859 26491126 PMC4656903

[49] Avila-Nava A Noriega LG Tovar AR Granados O Perez-Cruz C Pedraza-Chaverri J . Food combination based on a pre-hispanic Mexican diet decreases metabolic and cognitive abnormalities and gut microbiota dysbiosis caused by a sucrose-enriched high-fat diet in rats. Mol Nutr Food Res. 2017;61(1). doi: 10.1002/mnfr.201501023 27352915

[50] Sánchez-Tapia M Aguilar-López M Pérez-Cruz C Pichardo-Ontiveros E Wang M Donovan SM . Nopal (*Opuntia ficus indica*) protects from metabolic endotoxemia by modifying gut microbiota in obese rats fed high fat/sucrose diet. Sci Rep. (2017) 7:4716. doi: 10.1038/s41598-017-05096-4 28680065 PMC5498631

[51] FAO. The State of the World's Biodiversity for Food and Agriculture. Rome: FAO. (2019). Available online at: https://openknowledge.fao.org/server/api/core/bitstreams/50b79369-9249-4486-ac07-9098d07df60a/content (Accessed January 2, 2026).

[52] Grof-Tisza P Muller MH Gónzalez-Salas R Bustos-Segura C Benrey B. The Mesoamerican Milpa agroecosystem fosters greater arthropod diversity compared to monocultures. Agric Ecosyst Environ. (2024) 372:109074. doi: 10.1016/j.agee.2024.109074

[53] González-Esquivel CE Briones-Guzmán C Tovar-López E . Sustainability evaluation of contrasting Milpa systems in the Yucatán Peninsula, Mexico. Environ Dev Sustain. (2025) 27:9233–55. doi: 10.1007/s10668-023-04281-y

[54] De Cosmi V Mazzocchi A Milani GP Agostoni C. Dietary patterns vs. dietary recommendations. Front Nutr. (2022) 9:883806. doi: 10.3389/fnut.2022.883806 35592632 PMC9113217

[55] Rodríguez-Robayo KJ Méndez-López ME Molina-Villegas A Juárez L. What do we talk about when we talk about Milpa? A conceptual approach to the significance, topics of research and impact of the mayan Milpa system. J Rural Stud. (2020) 77:47–54. doi: 10.1016/j.jrurstud.2020.04.029

[56] Fernández-Demeneghi R Sánchez-Bizama J Martinez-Moreno AG Vargas Moreno I Ramirez-Rodriguez R Puga Olguín A . Mindful eating as the next therapeutic frontier in nutritional psychiatry. Front Nutr. (2026) 13:1726847. [Preprint] doi: 10.3389/fnut.2026.1726847 41684776 PMC12890638

[57] Kasali FM Tusiimire J Kadima JN Agaba AG. Ethnomedical uses, chemical constituents, and evidence-based pharmacological properties of *Chenopodium ambrosioides* L.: extensive overview. Future J Pharm Sci. (2021) 7:153. doi: 10.1186/s43094-021-00306-3

[58] Segura-Campos MR Ruiz-Ruiz JC Chel-Guerrero LA Betancur-Ancona DA. Capsicum chinense: composition and functional properties. In:Kristberg Kristbergsson Semih Ötles, editors. Functional Properties of Traditional Foods. Integrating Food Science and Engineering Knowledge Into the Food Chain Vol 12. New York: Springer. (2016). p. 289–92. doi: 10.1007/978-1-4899-7662-8_20

[59] Santiago-López L García HS González-Córdova AF Vallejo-Córdoba B Hernández-Mendoza A. Antioxidant and anti-inflammatory properties of huauzontle (*Chenopodium berlandieri* subsp. nuttalliae, Chenopodiaceae) fermented by *Lactiplantibacillus plantarum* L p22. Acta Bot Mex. (2023) 130:e2161. doi: 10.21829/abm130.2023.216160

[60] El-Mostafa K El Kharrassi Y Badreddine A Andreoletti P Vamecq J El Kebbaj MHS . Nopal cactus (*Opuntia ficus-indica*) as a source of bioactive compounds for nutrition, health and disease. Molecules. (2014) 19:14879–901. doi: 10.3390/molecules19091487968 25232708 PMC6270776

[61] Valdez-Morales M Céspedes-Carlos L Valverde ME Ramírez-Chávez E Paredes-López O. Phenolic compounds, antioxidant activity and lipid profile of huitlacoche mushroom (*Ustilago maydis*) produced in several maize genotypes at different stages of development. Plant Foods Hum Nutr. (2016) 71:436–43. doi: 10.1007/s11130-016-0572-3 27605221

[62] Ali MY Sina AAI Khandker SS Neesa L Tanvir EM Kabir A . Nutritional composition and bioactive compounds in tomatoes and their impact on human health and disease: a review. Foods. (2021) 10:45. doi: 10.3390/foods10010045 33375293 PMC7823427

[63] Shenstone E Lippman Z Van Eck J. A review of nutritional properties and health benefits of *Physalis* species. Plant Foods Hum Nutr. (2020) 75:316–25. doi: 10.1007/s11130-020-00821-3 32385801

[64] Di Lorenzo R Castaldo L Sessa R Ricci L Vardaro E Izzo L . Chemical profile and promising applications of *Cucurbita pepo* L. flowers. Antioxidants (Basel). (2024) 13:1476. doi: 10.3390/antiox13121476 39765805 PMC11673392

[65] Vieira EF Pinho O Ferreira IMPLVO Delerue-Matos C. Chayote (*Sechium edule*): a review of nutritional composition, bioactivities and potential applications. Food Chem. (2019) 275:557-68. doi: 10.1016/j.foodchem.2018.09.146 30724233

[66] García-Vásquez R Vera-Guzmán AM Carrillo-Rodríguez JC Pérez-Ochoa ML Aquino-Bolaños EN Alba-Jiménez JE . Bioactive and nutritional compounds in fruits of pepper (*Capsicum annuum* L.) landraces conserved among indigenous communities from Mexico. AIMS Agric Food. (2023) 8:832–50. doi: 10.3934/agrfood.2023044

[67] Mateos-Maces L Chávez-Servia JL Vera-Guzmán AM . Edible leafy plants from Mexico as sources of antioxidant compounds, and their nutritional, nutraceutical and antimicrobial potential: a review. Antioxidants (Basel). (2020) 9:531. doi: 10.3390/antiox9060541 32575671 PMC7346153

[68] Ghorani V Saadat S Khazdair MR Gholamnezhad Z El-Seedi H Boskabady MH. Phytochemical characteristics and anti-inflammatory, immunoregulatory, and antioxidant effects of *Portulaca oleracea* L.: a comprehensive review. Evid Based Complement Alternat Med. (2023) 2023:2075444. doi: 10.1155/2023/207544475 37693918 PMC10484659

[69] Mannino G Serio G Bertea CM Chiarelli R Lauria A Gentile C. Phytochemical profile and antioxidant properties of the edible and non-edible portions of black sapote (*Diospyros digyna* Jacq.). Food Chem. (2022) 380:132137. doi: 10.1016/j.foodchem.2022.132137 35093655

[70] Sánchez-Recillas E Campos-Vega R Pérez-Ramírez IF Luzardo-Ocampo I Cuéllar-Núñez ML Vergara-Castañeda HA. Garambullo (*Myrtillocactus geometrizans*): effect of in vitro gastrointestinal digestion on the bioaccessibility and antioxidant capacity of phytochemicals. Food Funct. (2022) 13:4699–713. doi: 10.1039/D1FO04392G 35380561

[71] de Oliveira AC Moreira Mar J Frota Corrêa R Sanches EA Campelo PH Ramos AS . Pouteria spp. fruits: health benefits of bioactive compounds and their potential for the food industry. Food Res Int. (2023) 164:113310. doi: 10.1016/j.foodres.2023.113310 37803621

[72] Barreca D Laganà G Ficarra S Tellone E Leuzzi U Galtieri A . Evaluation of the antioxidant and cytoprotective properties of the exotic fruit *Annona cherimola* Mill. (*Annonaceae*). Food Res Int. (2011) 44:2302–10. doi: 10.1016/j.foodres.2011.02.031

[73] Nishikito DF Borges ACA Laurindo LF Otoboni AMMB Direito R Goulart RA . Anti-inflammatory, antioxidant, and other health effects of dragon fruit and potential delivery systems for its bioactive compounds. Pharmaceutics. (2023) 15:159. doi: 10.3390/pharmaceutics15010159 36678789 PMC9861186

[74] Yahia EM. Gutiérrez-Orozco F, Arvizu-de León C. Phytochemical and antioxidant characterization of mamey (*Pouteria sapota* Jacq HE Moore & Stearn) fruit. Food Res Int. (2011) 44:2175–81. doi: 10.1016/j.foodres.2010.11.029

[75] Nazhand A Lucarini M Durazzo A Zaccardelli M Cristarella S Souto SB . Hawthorn (*Crataegus* spp.): An updated overview on its beneficial properties. Forests. (2020) 11:564. doi: 10.3390/f1105056476

[76] Kong YR Jong YX Balakrishnan M Bok ZK Weng JKK Tay KC . Beneficial role of Carica papaya extracts and phytochemicals on oxidative stress and related diseases: a mini review. Biology. (2021) 10:287. doi: 10.3390/biology1004028777 33916114 PMC8066973

[77] Ramírez-Rodríguez Y Ramírez V Robledo-Márquez K García-Rojas N Rojas-Morales P Arango N . Stenocereus huastecorum-fruit juice concentrate protects against cisplatin-induced nephrotoxicity by nitric oxide pathway activity and antioxidant and antiapoptotic effects. Food Res Int. (2022) 160:111337. doi: 10.1016/j.foodres.2022.11133778 36076365

[78] Sameh S Al-Sayed E Labib RM Singab AN. Genus spondias: a phytochemical and pharmacological review. Evid Based Complement Alternat Med. (2018) 2018:5382904. doi: 10.1155/2018/538290483 29785194 PMC5896409

[79] Bangar S Sharma N Kaur H Kaur M Sandhu KS Maqsood S . A review of Sapodilla (*Manilkara zapota*) in human nutrition, health, and industrial applications. Trends Food Sci Technol. (2022) 127:319–34. doi: 10.1016/j.tifs.2022.05.016

[80] Morales P. Ramírez-Moreno E, Sánchez-Mata MC, Carvalho AM, Ferreira ICR. Nutritional and antioxidant properties of pulp and seeds of two *Xoconostle cultivars* (*Opuntia joconostle* FAC Weber ex Diguet and Opuntia matudae Scheinvar) of high consumption in Mexico. Food Res Int. (2012) 46:279–85. doi: 10.1016/j.foodres.2011.12.031

[81] Coria-Téllez AV Montalvo-González E Yahia EM Obledo-Vázquez EN. Annona muricata: A comprehensive review on its traditional medicinal uses, phytochemicals, pharmacological activities, mechanisms of action and toxicity. Arab J Chem. (2018) 11:662–91. doi: 10.1016/j.arabjc.2016.01.004

[82] Cabral B Siqueira EMS Bitencourt MAO Lima MCJS Lima AK Ortmann CF . Phytochemical study and anti-inflammatory and antioxidant potential of Spondias mombin leaves. Rev Bras Farmacogn. (2016) 26:304–11. doi: 10.1016/j.bjp.2016.02.002

[83] Singhania N Kumar R Pramila Bishnoi S Ray AB Diwan A. Bioactive properties and health benefits of amaranthus. In: Harvesting Food from Weeds. (2023). p. 351–83. doi: 10.1002/9781119793007.ch1084

[84] Grancieri M Martino HSD Gonzalez de Mejia E. Chia seed (*Salvia hispanica* L.) as a source of proteins and bioactive peptides with health benefits: a review. Compr Rev Food Sci Food Saf . (2019) 18:480–99. doi: 10.1111/1541-4337.1242385 33336944

[85] Adeleke BS Babalola OO. Oilseed crop sunflower (*Helianthus annuus*) as a source of food: Nutritional and health benefits. Food Sci Nutr. (2020) 8:4666–84. doi: 10.1002/fsn3.178386 32994929 PMC7500752

[86] Ishaq S Jafri L. Biomedical importance of cocoa (*Theobroma cacao*): significance and potential for the maintenance of human health. Matrix Sci Pharma. (2017) 1:1–5. doi: 10.26480/msp.01.2017.01.0587

[87] Çiftçi S Suna GÜ. Functional components of peanuts (*Arachis hypogaea* L.) and health benefits: a review. Future Foods. (2022) 5:100140. doi: 10.1016/j.fufo.2022.10014097

[88] Turco I Ferretti G Bacchetti T. Review of the health benefits of Faba bean (*Vicia faba* L.) polyphenols. J Food Nutr Res. (2016) 55:283–93.

[89] Aslani Z Alipour B Mirmiran P Bahadoran Z. Lentil's (*Lens culinaris* L.) functional properties in prevention and treatment of non-communicable chronic diseases: a review *Int J Nutr Food Sci*. (2014) 4:15–20. doi: 10.11648/j.ijnfs.s.2015040201.14

[90] Fonseca Duarte P Alves Chaves M Dellinghausen Borges C Barboza Mendonça CR. Avocado: characteristics, health benefits and uses. Ciênc Rural. (2016) 46:747–54. doi: 10.1590/0103-8478cr20141516

[91] Fernández-Sánchez F García-Barradas O Mendoza-López MR Juárez-Trujillo N Luna-Solano G Romero-Luna HE . Nutritional potential of dehydrated *Sphenarium rugosum* powder: an insight into fatty acids and minerals. Food Chem. (2025) 485:144489. doi: 10.1016/j.foodchem.2025.144489 40319589

[92] Kishimoto Y Sugihara N. Egg consumption and human health: a comprehensive review of the effects on serum lipids, antioxidant status, and cardiovascular outcomes. J Poult Sci. (2026) 63:2026001. doi: 10.2141/jpsa.2026001 41497962 PMC12765572

[93] Connolly G Clark CM Campbell RE Byers AW Reed JB Campbell WW. Poultry consumption and human health: how much is really known? a systematically searched scoping review and research perspective. Adv Nutr. (2022) 13:2115–24. doi: 10.1093/advances/nmac074 36351778 PMC9776623

[94] Chen J Jayachandran M Bai W Xu B. A critical review on the health benefits of fish consumption and its bioactive constituents. Food Chem. (2022) 369:130874. doi: 10.1016/j.foodchem.2021.130874 34455321

[95] Mohidin S Moshawih S Hermansyah A Asmuni MI Shafqat N Ming LC. Cassava (*Manihot esculenta* Crantz): a systematic review for the pharmacological activities, traditional uses, nutritional values, and phytochemistry. J Evid-Based Integr Med. (2023) 28:2515690X231206227. doi: 10.1177/2515690X231206227 37822215 PMC10571719

[96] Alam MK. A comprehensive review of sweet potato (*Ipomoea batatas* [L.] Lam): Revisiting the associated health benefits Trends. Food Sci Technol. (2021) 115:512–29. doi: 10.1016/j.tifs.2021.07.001

[97] Cianciosi D Forbes-Hernández TY Afrin S Gasparrini M Reboredo-Rodriguez P Manna PP . Phenolic compounds in honey and their associated health benefits: a review. Molecules. (2018) 23:2322. doi: 10.3390/molecules23092322 30208664 PMC6225430

